# Exposure region of the Kawase approach and its correlation with skull base anatomy: An evaluation with digital models

**DOI:** 10.3389/fsurg.2022.1047949

**Published:** 2023-01-06

**Authors:** Yong Yan, Tao Xu, Yuqing Zhao, Qiyong Mei, Lei Jiang, Lijun Hou

**Affiliations:** Department of Neurosurgery, Changzheng Hospital, Naval Medical University, Shanghai, China

**Keywords:** transpetrosal approach, 3D reconstruction, skull base, computational anatomy, quantitative, surgical simulation

## Abstract

The Kawase approach is one of the most used trajectories in skull base surgery. The exposure range of the approach and its correlation with skull base anatomy still demand more exploration. With the help of digital rebuilding, analysis, and measurement, we evaluated the exposure range of the Kawase and extended Kawase approaches and analyzed the correlation between the exposure range and the variants of the petrosal and clival anatomy. The finding of the study demonstrated that compared to the sub-temporal approach, the Kawase approach and the extended Kawase approach significantly added the exposure range in the upper, middle, and partial inferior regions of the clivus. The gains in the exposure volume and area are more when the manipulation angle is less than 135°.

## Introduction

The Kawase approach is a further development of the sub-temporal approach by removing the bones in the petrosal apex. It was described by Kawase et al. in 1985 (1). The approach overcomes the obstructions of the petrosal range and extends the region exposed in the posterior cranial fossa without bringing more retraction of the temporal lobe and Labei's vein (2). By this approach, many lesions in the petrosal apex and the upper and middle clivus could be well exposed and safely resected, including meningioma, chordoma, basilar trunk aneurysm, prepontine epidermoid, trigeminal schwannoma, and pontine cavernoma (1, 3–5).

In the standard Kawase approach, the drilling range of the bone is restricted to the petrosal apex, with a rhomboid shape by the view from the lateral-superior direction. The boundaries of the rhomboid are defined by the arcuate eminence posteriorly, the greater superficial petrosal nerve or petrosal segment of the internal carotid artery laterally, the posterior edge of the trigeminal nerve anteriorly, and the petrosal ridge medially (6, 7). The deep boundary of debone, which is also the boundary between the petrosal apex and clivus, is restricted. The Kawase approach could be further extended by drilling bones beyond IPS. It could expand the deboning range to the upper and middle clivus and the jugular tubercle (JT) (8–10). Endoscopic assistance could extend the drilling range further to the condyle (8). However, from a microscopic view, further deboning beyond the JT will be difficult.

The Kawase approach is one of the most used trajectories in skull base surgery. With the help of digital rebuilding, analysis, and measurement, we evaluated the exposure range of the Kawase and extended Kawase approaches and also analyzed the correlation between the exposure range and the variants of the petrosal and clival anatomy.

## Materials and methods

### Data processing and rebuilding

**Figure 1:**
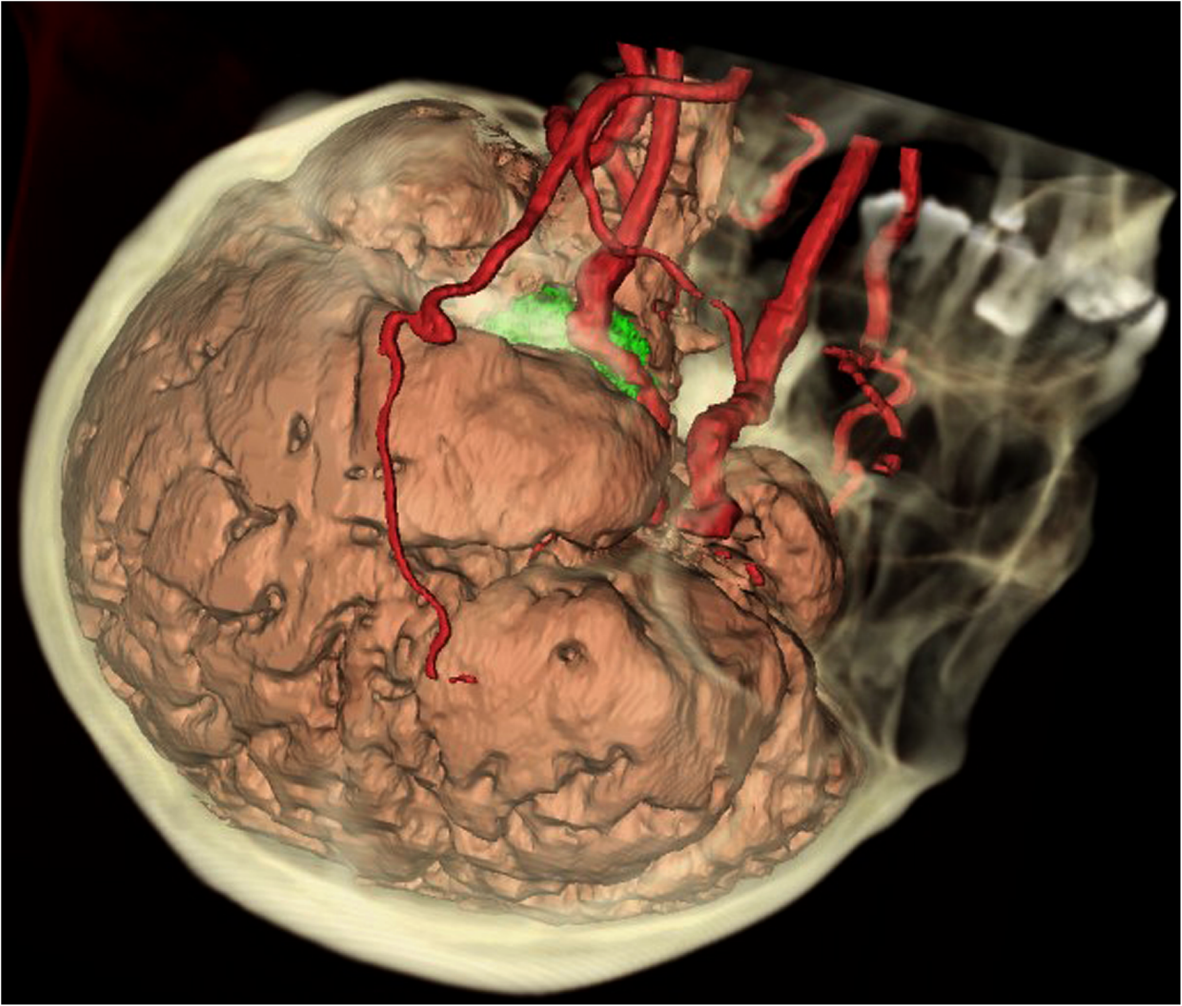
Integrated 3D model of the brain, vessels, and bones. The skull (yellow), the cerebral arteries (red), and the brain (pink yellow) were rebuilt separately. A tumor (green) could be seen in the petroclival region, surrounded by the skull base bones, the vessels, the cerebral lobes, and the brain stem.

The data of thin-cut CT scans of six patients in the DICOM format was collected for analysis. Materialise Mimics 21.0 and 3-matic Research 13.0 (Materialise, Leuven, Belgium) software packages were used for data rebuilding, processing, and measurement. The skull bone, cerebral arteries, and brain tissue were rebuilt separately (Figure 1). Critical anatomic structures relevant to the Kawase approach were marked with cylinder, linear, or point objects, including the inner acoustic canal, the cochlea, the trigeminal nerve, the superior petrosal sinus, IPS, the glomus jugulare, and JT.

### Simulation of the sub-temporal, the Kawase, and the extended Kawase approaches

A bone widow with a size of 6 cm*4 cm was made in the temporal squamous. The lower edge of the bone window was made nearly flat to the bottom of the middle skull fossa. To simulate the intraoperational limits for brain retraction, a plane was placed in the temporal region with a slope angle of 20°to the horizontal level and with a distance of 2.5 cm from the lower edge of the bone window. There is evidence supporting that a retraction of the temporal lobe within 2.5 cm and 20° from the skull base is safe during surgery (11–14). To protect the labei's vein, the posterior temporal lobe should avoid retraction as much as possible (15). To simulate this surgery step, another slope plane was placed on the posterior part of the temporal bone window. Then, the mask was calculated and saved for the following analysis.

To simulate the Kawase approach, the petrosal apex bone was removed with the “ellipse erase” function in the “edit mask” panel. The boundaries of the removal bone were defined by the following: (1) laterally, the petrosal segment of the internal carotid artery; (2) lateral-posteriorly, the arcuate eminence; and (3) inferior-medially, the IPS. The cochlea was skeletonized in the angle formed by the petrous carotid artery and the inner acoustic canal. The bones under the root of the trigeminal nerve could be drilled by adjusting the direction of exposure from the posterior, which was still within the permits of the retraction planes mentioned above. After the removal of the bone, the inner acoustic canal could be seen in the posterior part of the drilling range of the petrosal apex. Almost the whole length of the IPS could be exposed from its origin in the ridge of the petrous bone to its ending in the jugular foramen (Supplementary Video).

In the extended Kawase approach, the bone deeper to the IPS was further drilled. Drilling beyond the upper and middle IPS could extend the resection of the bone to the upper and middle clivus, and drilling beyond the lower IPS could partly or totally remove the JT.

### Exposure evaluation

To analyze the exposure ranges of the subtemporal, Kawase, and extended Kawase approaches, the intracranial spaces were filled with contents with soft tissue signals. Then, the tissues were simulated for resection within the exposure limits of each approach and the limits of the retraction planes. The residual tissue after simulated resection signifies the exposure limit of each approach. Spline lines were used to delineate the edge of the residual tissue, which signifies the exposure limits of each approach on the skull base. Parameters including the volumes of the residual tissues, the areas surrounded by the spline lines, and the distances of the spline lines to the edge of the foramen magnum were calculated. The midline is the line between the basion and the crista galli, and the para-middle line is the line parallel to the midline and across the anterior edge of the JT.

### Measurement and analysis

**Figure 2:**
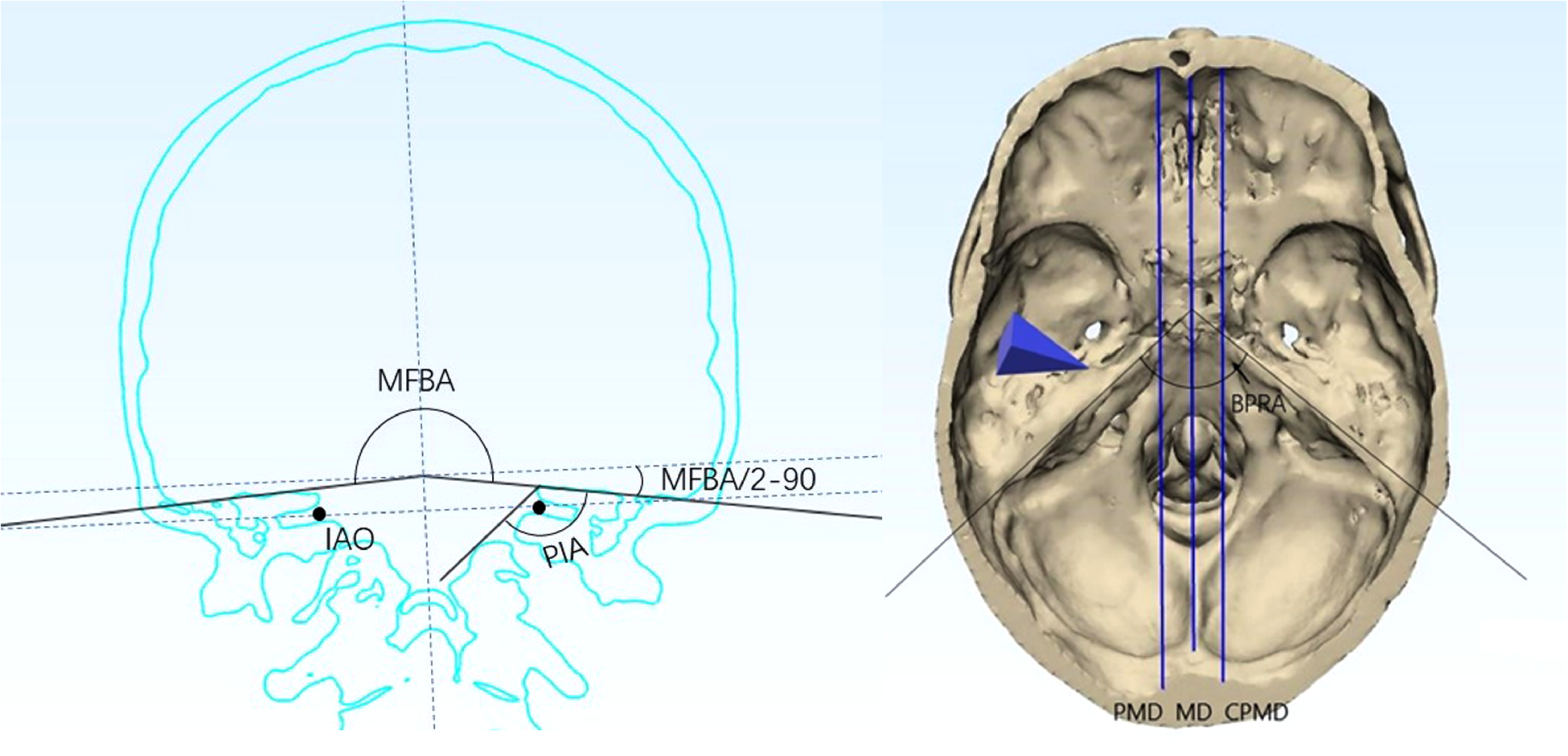
Definition of angles and lines set in the model: (**A**) MFBA and PIA; (**B**) BPRA, PMD, MD, and CPMD. BPRA, bilateral petrous ridge angle; PIA, petrous inner angle; MFBA, middle fossa bottom angle; MD, midline; PMD, para-middle line; CPMD, contralateral para-middle line.

By the measuring panels in software, the following parameters were gauged in separate cases (Figure 2).

(1) Angle measurement:
  • The bilateral petrous ridge angle (BPRA): the angle formed by the two petrous ridges.
  • The petrous inner angle (PIA): the angle measured at the coronal section across the inner acoustic orifice (IAO); one arm of the angle is the bottom of the middle fossa, and the other arm is formed by the line between the tip of the petrosal ridge and the protuberance near the JT. The coronal section is defined as the plane vertical to the line across the upper point of the external ear canal and the inferior point of the obit.
  • The middle fossa bottom angle (MFBA): the angle formed by bilateral planes of the middle cranial fossa at the section across the IAO.
  • The manipulation angle (MA) was defined as follows:
    MA=PIA−(MFBA/2−90)
(2) Volume measurement:
  • Residual volume by the subtemporal approach: the volume of the residual tissues after simulated resection of the subtemporal approach.
  • Residual volume by the Kawase approach: the volume of the residual tissues after simulated resection of the Kawase approach.
  • Residual volume by the extended Kawase approach: the volume of the residual tissues after simulated resection of the extended Kawase approach.
(3) Area measurement:
  • Residual area by the subtemporal approach: the area delineated by spline lines after simulated resection of the subtemporal approach.
  • Residual area by the Kawase approach: the area delineated by spline lines after simulated resection of the Kawase approach.
  • Residual area by the extended Kawase approach: the area delineated by spline lines after simulated resection of the extended Kawase approach.
(4) Distance measurement:
  • Midline distance (MD): the distance between the basion and the spline line in the middle plane (vertical plane that is across the midline).
  • Para-middle distance (PMD): the distance between the edge of the foramen magnum and the spline line in the para-middle plane (vertical plane that is across the para-middle line).
  • Para-middle distance in the contra-side (CPMD): the distance between the edge of the foramen magnum and the spline line in the contralateral para-middle plane.
(5) Statistical analysis

Linear regression and paired *t*-test were used to analyze the data, with significance set at *P* < 0.05 (in figures, *P* < 0.05 is marked as *; *P* < 0.01 is marked as **; and *P* < 0.001 is marked as ***). Statistical data were analyzed by Excel 2013 (Microsoft, United States) and SPSS 26.0 (IBM, United States).

## Results

**Figure 3:**
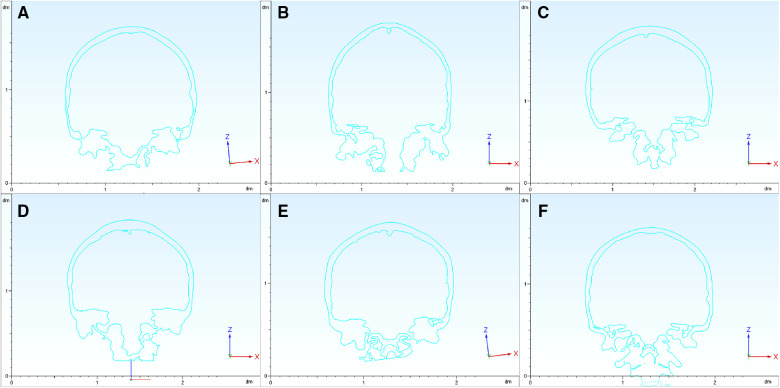
Coronal section of the skull. The coronal sections across the bilateral IAO show varied shapes among the six cases (panels **A**–**F** signify cases 1–6, respectively). PIA and MFBA are measured based on these coronal sections. IAO, inner acoustic orifice; PIA, petrous inner angle; MFBA, middle fossa bottom angle.

The coronal section across the bilateral IAO showed varied shapes among cases (Figure 3). The range of PIA was between 127.8° and 143.4°. In a certain case, the PIA on the bilateral sides could be different, and the biggest difference was 8.5°. The range of MFBA was between 168.6° and 197.8°. The bottom of the middle cranial fossa lifts more when MFBA gets larger. There was no significant relationship between BPRA and other angle parameters such as PIA, MFBA, or MA (*P* > 0.05) (Table 1).

**Table 1:** General information.

Case	Side	Gender	Age	BPRA	PIA	MFBA	MA
1	R	m	53	114.8	135.7	168.6	141.4
	L				133.8		139.5
2	R	m	53	99.9	143.4	169.5	148.6
	L				138.5		143.7
3	R	m	63	117.7	131.2	197.8	122.3
	L				128.5		119.6
4	R	m	74	105.7	136.3	175.4	138.6
	L				127.8		130.1
5	R	f	66	101.9	138.6	175.9	140.7
	L				137.8		139.8
6	R	m	66	104.3	138.5	185.0	136.0
	L				131.3		128.8

BPRA, bilateral petrous ridge angle; PIA, petrous inner angle; MFBA, middle fossa bottom angle; MA, manipulation angle; m, male; f, female.

**Figure 4:**
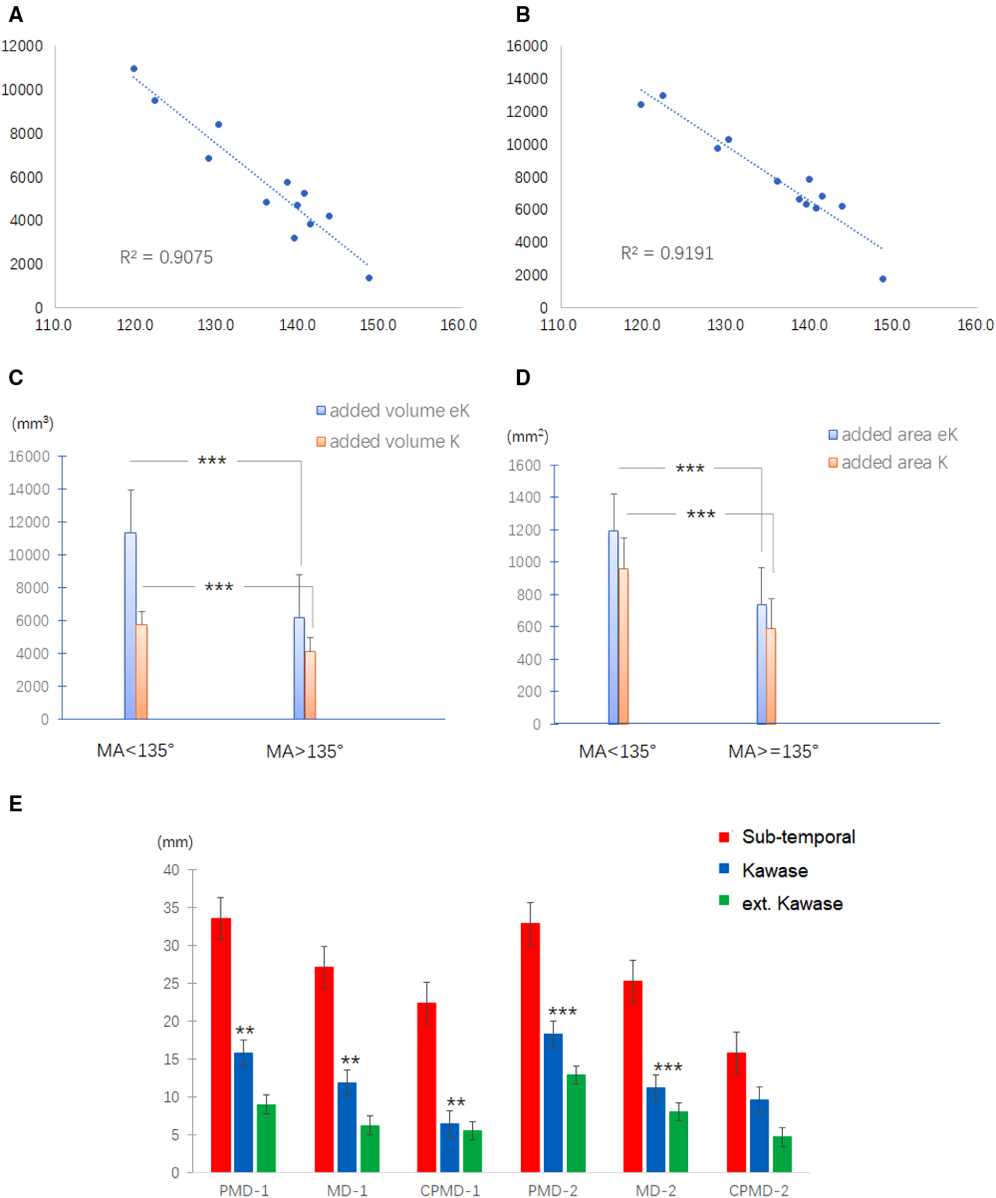
Correlation between the exposure range and the skull base anatomy. (**A,B**) Relationship between MA and added exposure volume for Kawase (**A**) and extended Kawase (**B**) approaches. (**C,D**) Exposure gains in the volume are higher in the Kawase approach or extended Kawase approach in group 1 (MA < 135°) than those in group 2 (MA ≥ 135°) (*P* < 0.01) (**C**). Similarly, the added exposure areas are larger in the Kawase or extended Kawase approach when MA is less than 135°(*P* < 0.01) (**D**). (**E**) Definitions of MD, PMD, and CPMD. The midline is the line between the basion and the crista galli; the para-middle line is the line parallel to the midline and across the anterior edge of the JT (MD, midline; PMD, para-middle line; CPMD, contralateral para-middle line). (**F**) Inferior limits for subtemporal (red column), Kawase (blue column), and extended Kawase (green column) approaches in MD, PMD, and CPMD of group 1 (MA < 135°) and group 2 (MA ≥ 135°). MA, manipulation angle.

MA is a parameter designed to combine the effects of PIA and MFBA on the manipulation corridor. Compared with the subtemporal approach, the exposure gain for the Kawase or extended Kawase approach was found to be inversely related to MA. An inverse linear relationship was found between MA and added volume for the Kawase approach (*R*^2 ^= 0.9075, *P* < 0.001, Figure 4A) and extended Kawase approach (*R*^2 ^= 0.9191, *P* < 0.001, Figure 4B). We further classified the data into two groups based on if the MA was smaller than 135° (group 1: MA < 135°; group 2: MA ≥ 135°). Compared with the subtemporal approach, the exposure gains in the volume are higher in the Kawase approach or extended Kawase approach in group 1 than those in group 2 (*P* < 0.001) (Figure 4C). The added volumes are 5,736 ± 1,497 mm^3^ and 4,141 ± 1,290 mm^3^ for the Kawase approach and 11,346 ± 1,585 mm^3^ and 6,179 ± 1,913 mm^3^ for the extended Kawase approach in group 1 and group 2, respectively. Similarly, the added exposure areas are larger in the Kawase or extended Kawase approach when MA is less than 135°(*P* < 0.001) (Figure 4D). The added areas are 961 ± 114 mm^2^ and 588 ± 118 mm^2^ for the Kawase approach and 1,192 ± 116 mm^2^ and 737 ± 99 mm^2^ for the extended Kawase approach in groups 1 and 2, respectively (Table 2).

**Table 2:** Added volume and area in Kawase and extended Kawase approaches (mm^3^/mm^2^).

	Added volume in Kawase	Added volume in extended Kawase	Added area in Kawase	Added area in extended Kawase
MA < 135°	5,736 ± 1,497	11,346 ± 1,585	961 ± 114	1,192 ± 116
MA ≥ 135°	4,141 ± 1,290	6,179 ± 1,913	588 ± 118	737 ± 99

MA, manipulation angle.

**Figure 5:**
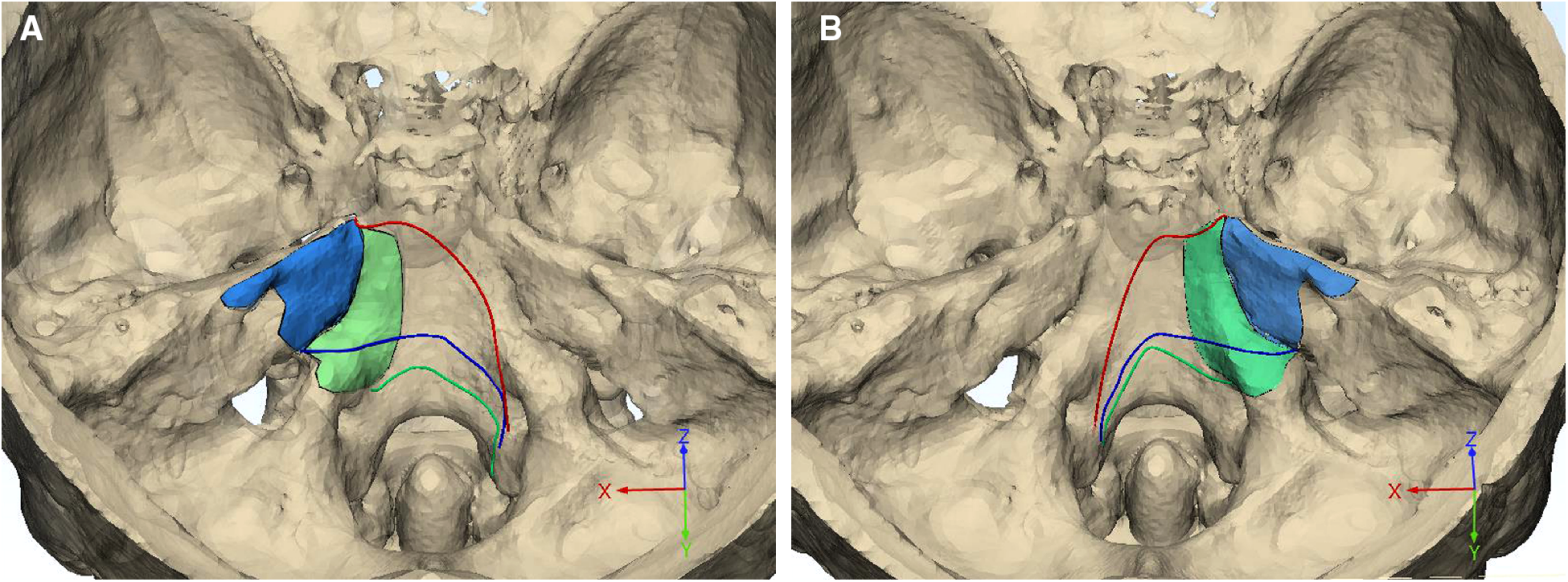
Exposure limits of different approaches. The inferior limits for subtemporal (red line), Kawase (blue line), and extended Kawase (green line) approaches are delineated in the skull base of group 1 (A, MA < 135°) and group 2 (B, MA ≥ 135°). The debone ranges for the Kawase (blue area) and extended Kawase (green area) approaches are also marked. MA, manipulation angle.

To delineate the exposure range in the cranial side of the clivus and petrous, the inferior limits for each approach were measured in the middle and para-middle lines (the lines of MD, PMD, and CPMD, see Figure 2B). It was found that the Kawase approach has pushed the exposure limit nearer to the occipital fossa edge than the subtemporal approach. Similarly, the limit of the extended Kawase approach was lower than that of the Kawase approach (Table 3 and Figure 4E). To make it more directly perceived, the limits for each approach were delineated in the skull base in group 1 and group 2 (Figure 5). It could be seen that the added exposure areas for the Kawase approach and the extended Kawase approach were wider in group 1 than those in group 2. The result was consistent with the added areas of these approaches in the above analysis.

**Table 3:** Exposure limits in the middle and para-middle lines among different approaches (mm).

	Group 1: MA < 135°			Group 2: MA ≥ 135°		
	PMD	MD	CPMD	PMD	MD	CPMD
Subtemporal	33.6 ± 4.0	27.1 ± 4.1	22.4 ± 6.1	32.9 ± 3.5	25.3 ± 4.4	15.8 ± 6.3
Kawase	15.8 ± 3.5	11.9 ± 5.6	6.4 ± 1.8	18.3 ± 5.2	11.2 ± 3.2	9.6 ± 6.1
Extended Kawase	9.0 ± 5.1	6.3 ± 3.3	5.6 ± 2.4	12.9 ± 4.7	8.1 ± 3.9	4.7 ± 2.4

MA, manipulation angle; PMD, para-middle distance; MD, middle distance; CPMD, para-middle distance in the contra-side.

## Discussion

### Importance of the accurate evaluation of the exposure range and influence factors of the skull base approaches

The operation of the petrosal–clival lesion is still a great challenge in the neurosurgical field. The surgical difficulties are mainly caused by the following reasons. First, the petrosal–clival region lies almost at the center of the skull base. Its deep position makes it very difficult to get. Second, there are many critical structures surrounding the region, including the arteries (the petrosal and cavernous segments of the internal carotid artery and the basal and vertebral arteries); the III–XI cranial nerves, the tympanum, the cochlea, the semicircular canals, the dura sinuses (the cavernous sinus, the sigmoid sinus, the superior petrosal sinus, and IPS), the brain stem, and the cerebellum. Third, there are many bony ridges or protuberances surrounding the area, which need to be removed for exposure. In addition to the Kawase approach, there are many other approaches that could be chosen for treating lesions of the site, including the transcavernous approach (16), the presigmoidal transpetrosal approach, the suboccipital postsigmoidal approach (17), and the extended endoscopic transsphenoidal–transclival approach (9). However, the factors mentioned above have brought difficulties in getting to the lesions, whatever approach is chosen. Therefore, the accurate evaluation of the exposure range of the approaches is necessary to help the surgeon choose a proper approach specialized to the site and range of a lesion. There could be other factors influencing the exposure range of an approach, for example, the position of the sigmoid sinus or the apophysis extent of the petrosal ridge. The influence of the anatomic variants on the exposure of the approaches is also important in deciding which one is more suitable for an individualized patient.

### Anatomic variants and their correlation with the exposure range of the transpetrosal approaches

**Figure 6:**
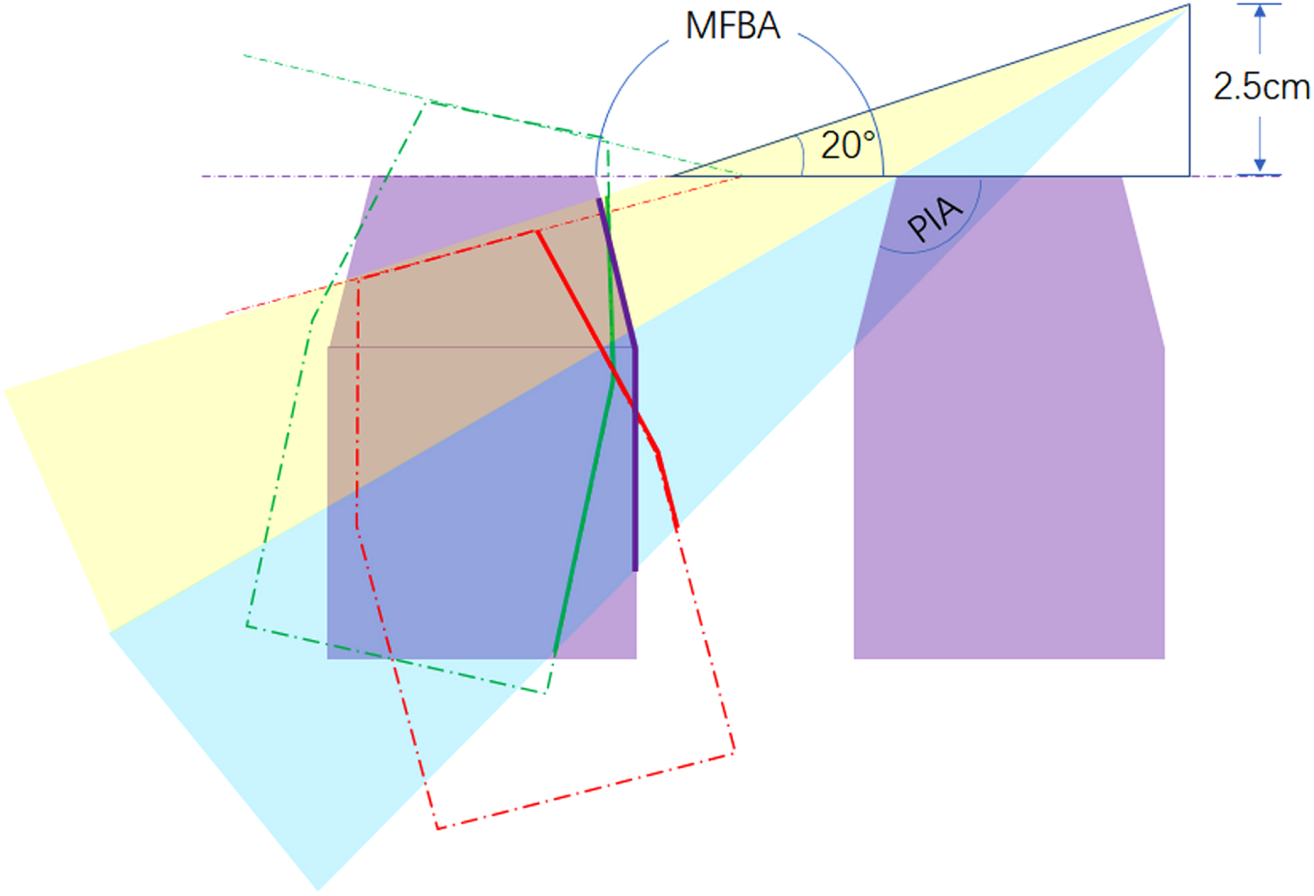
Effect of PIA and MFBA on the exposure of the Kawase or extended Kawase approach. PIA affects the range of bone removed by deboning the petrous apex and the sight angle added by the procedure (blue shade). MFBA affects the exposure range of the inner face of the petrosal bone in the contra side. When MFBA increases, the exposure range becomes smaller (purple line, MFBA = 180°; red line, MFBA > 180°; green line, MFBA < 180°). MA is a parameter designed to combine the effects of PIA and MFBA. PIA, petrous inner angle; MFBA, middle fossa bottom angle; MA, manipulation angle.

The main manipulation corridor for subtemporal, Kawase, and extended Kawase approaches surrounds the line across the bilateral IAO (18). The anatomic variants in the coronal section across the bilateral IAO play an important role in the exposure of transpetrosal approaches. PIA affects the perspective angle added by drilling the petrous apex. If PIA reduces, the shape of the petrosal apex becomes steeper, and the added perspective angle increases by drilling the petrosal apex (Figure 6). MDBA affects the exposure area of the contralateral petrosal and occipital bones. As shown in Figure 5, if MDBA is larger than 180°, the contralateral petrosal ridge becomes relatively lower in perspective, and the exposure area decreases. MA is a parameter combining the effects of PIA and MFBA. As proven by the results of the current study, MA shows an inverse linear correlation with the added exposure of the Kawase or extended Kawase approach. By dividing the cases into two groups based on whether MA is less than 135°, the volume and area gained by removing the bone surrounding the petrosal apex will be larger in the group with MA < 135°. However, in either group, deboning the petrosal apex will increase the exposure of the upper and middle clivus.

### Critical areas and structures in the transpetrosal approaches

In our clinical experience, we found that some areas are difficult to expose or with a much higher risk of nerve or vessel injuries in the Kawase approach. The region beneath the trigeminal impression is one of them. In the simulated course of removing bone from the area, the manipulation corridor is quite narrow and close to the trigeminal nerve, which would increase the risk of the 5th nerve damage in real operations (19–21). The region is also near Dorello's canal. In most cases, there is a small impression just lateral to the dorsum sellae, which is the position of Dorello's canal and the beginning of the IPS (22, 23). Removing bone near the region will increase the risk of injuring the 6th nerve (19, 20). In the simulated surgery, Dorello's canal lies in the deepest point of the region beneath the trigeminal root from a posterior perspective.

Another structure that has important functions and is easy to injure is the cochlea. Cochlear damage leads to permanent hearing loss. It lies in the intersection of the petrosal carotid artery and the inner acoustic canal (24, 25). The area is narrow but essential for drilling deeper bones, including the middle-inferior clivus and the JT (26). Therefore, the skeleton of the cochlea is necessary for the extended Kawase approach.

The IPS is an important landmark in both the Kawase and extended Kawase approaches (19, 27). For the Kawase approach, the IPS is the terminal level of deboning. On the other hand, for the extended Kawase approach, IPS is just the beginning of deeper bone drilling. The superior and middle clivus lie in the deep of the upper and middle parts of IPS, and the JT lies in the inferior part of it. It should be noted that the basilar artery generally lies quite near the clivus, and there is a risk of arterial injury while drilling the clivus bone deeper into the IPS (28). As mentioned above, the superior end of IPS is related to Dorello's canal, and its inferior end is related to the jugular foramen. The JT lies roughly in the extending line across the zygomatic root and the cochlea. The distance between the projection of JT on IPS and the frontier edge of the jugular foramen is about 5.5 mm (4–7 mm). Drilling the inferior part of IPS may lead to turbulent bleeding and may cause injury to the lower cranial nerves in operation (29).

In the extended Kawase approach, drilling bone beneath the IPS would possibly cause turbulent venous bleeding, injure the basilar artery, and increase the risks of injuring the 5th, 6th, and 9th–11th cranial nerves. All the reasons mentioned above limit its use in real operations.

### Priority and shortcomings of the digital model in anatomic analysis

The digital module has many priorities in the anatomic study. Unlike cadaveric study, it does not need cadaveric specimens and anatomic laboratories, so it is more available and convenient. Important structures, including nerves, vessels, and bone structures, could be readily rebuilt or marked in the model. Sections across given levels could be precisely set to ensure standardization for further analysis among different models. Volumes, areas, angles, and distances can be accurately measured (30). In the course of surgery simulation, the three-dimensional perspective and rotation freedom of the models could give the researchers a good sense of space and help them to build a positional relationship among critical structures (31–33).

On the other hand, anatomic studies based on digital models have some shortcomings. Some structures, like the nerves and brain tissues, could not be precisely rebuilt, so these have to be marked or simulated in the models. Some details of the tissue, like texture and capability of displacement, could not be present in the module. Therefore, it requires the researchers to be more knowledgeable about the studied approach to design suitable landmarks and planes to overcome the defects mentioned above.

### Limitations of the study

The course of petrosal apex deboning and soft tissue removal is done manually, so it could not precisely be standardized among cases. The added volume and area measurement does not consider the block of the brain stem and the basilar artery and the mobilization capability of structures like nerves and vessels.

## Conclusion

Compared to the subtemporal approach, transpetrosal approaches (including the Kawase approach and the extended Kawase approach) could significantly add the exposure volume and area in the upper and middle clivus, as well as partial inferior clivus. The exposure volume and area gain more when MA is less than 135°.

## Data Availability

The raw data supporting the conclusions of this article will be made available by the authors without undue reservation.
